# Iodate induced toxic retinopathy: a case report

**DOI:** 10.1186/s40942-018-0130-2

**Published:** 2018-08-01

**Authors:** Y. C. Venu Gopal Reddy, Anuj Sharma, Adheesh P. Shetty, Syed Mohideen Abdul Kader

**Affiliations:** Aravind Eye Hospital, S.N. High Road, Tirunelveli, Tamil Nadu 627001 India

**Keywords:** Iodate, Toxic retinopathy, Foveal atrophy, Iodination of salt

## Abstract

**Background:**

Iodine deficiency is a common preventable cause of intellectual and developmental disabilities. Iodine in the form of potassium iodate is added to the common salt under the National Iodine Deficiency Disorder Control Program in India. Overdose of iodate can lead to retinal toxicity.

**Case presentation:**

We hereby present a case of a 34 year old male patient who presented to us 10 years following iodate ingestion. There was widespread outer retinal atrophy, foveal atrophy and sub-retinal fibrosis noted on fundus evaluation. The fundus fluorescein angiogram was suggestive of window defects while the scotopic electroretinogram showed diminished amplitude pointing towards a grave prognosis.

**Conclusions:**

Excessive ingestion of potassium iodate can lead to outer retinal atrophy due to its toxicity to the retinal pigment epithelium and photo-receptors. The degree of damage is dependent on the ingested dose.

## Background

Iodate is an essential micronutrient required daily at an RDA of 100–150 μg for normal human growth and development. Deficiency of iodine can cause physical and mental retardation, cretinism, abortions, still births, deaf mutism and goitre. In fact, Iodine deficiency worldwide is the leading preventable cause of intellectual and developmental disabilities [1].

337 districts of the 414 surveyed in India in 1962 were found to be endemic to iodine deficiency disorders (> 5% prevalence). National Goitre Control Program (1962) was initially launched and was later renamed as the National Iodine deficiency Disorder Control Program (NIDDCP) in 1992 [2]. Potassium iodate is used for iodine supplementation of salt under this national program.

The ingested iodate is metabolised to iodide and is taken up by the thyroid gland. Overdose of this ingested iodate is however toxic to the retinal pigment epithelium and the photoreceptors. We herewith describe a case of iodate toxicity who presented to us 10 years following iodate ingestion posing a diagnostic dilemma.

## Case report

A 34 year old male patient, a worker in the salt mines, presented to us complaining of diminished vision in both eyes since the past 10 years. He also complained of diminished night vision which was stationary and non-progressive. His vital parameters and general physical examination was noted to be within normal limits.

**Fig. 1:**
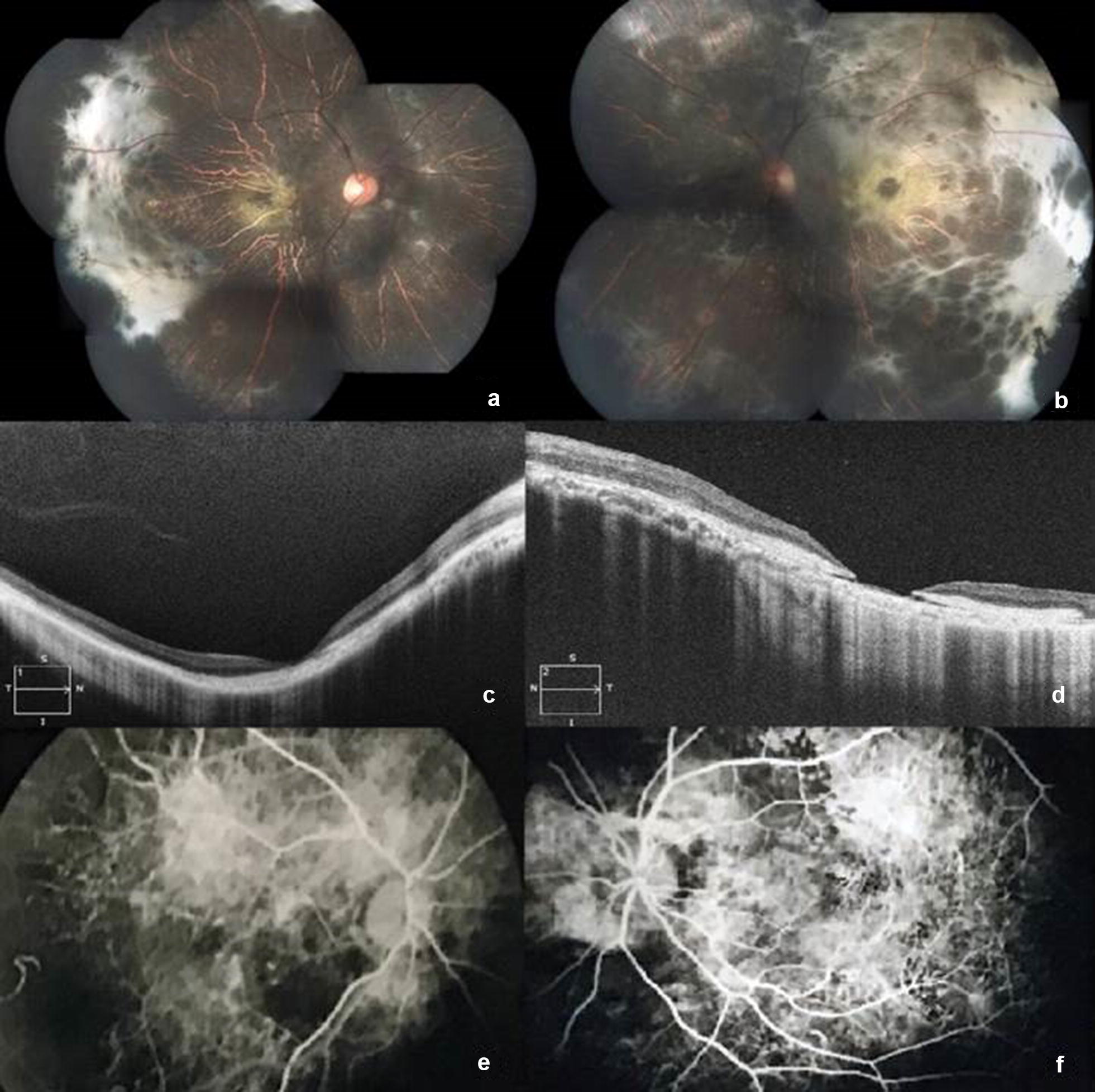
(**a**, **b**) The fundus evaluation revealed widespread outer retinal atrophy with visible large choroidal vessels. Marked temporal sub-retinal fibrosis was noted with foveal atrophy which was more marked in the left eye. (**c**, **d**) OCT revealed evidence of outer retinal atrophy with associated sub-foveal scarring. RPE discontinuity can be visualised in the left eye. (**e**, **f**) Fundus fluorescein angiogram noted a choroidal hyperfluorescence due to extensive windrow defects, suggestive of RPE loss

Ocular examination revealed a visual acuity of 6/36 (OD) and FCCF (OS). The pupillary reactions were sluggish in both eyes. The remainder of the anterior segment examination was noted to be normal. Fundus examination (Fig. 1a, b) showed presence of widespread outer retinal atrophy with visible choroidal vessels and peripheral sub-retinal scarring. The left eye shows evidence of foveal atrophy. A small peripheral rim of normal retina, around 1 disc diameter in size, was noted on indirect ophthalmoscopy.

We initially considered a diagnosis of choroideremia, retinitis pigmentosa and scarred posterior uveitis. There was no family history of nyctalopia and an evaluation of his siblings revealed a normal retina. The blood-work on uveitis also came out to be negative.

We once again reviewed the past medical history and upon detailed inquisition the patient divulged a history of a suicide attempt 10 years ago. He stated that he had consumed the chemical used in the iodination of salt after which he noted the diminished vision in both eyes.

We carried out an OCT evaluation (Fig. 1c, d) of the patient which showed evidence of outer retinal atrophy with associated sub-foveal scarring which was more pronounced in the left eye. A thinning of the choriocapillaris was evident in the OCT scans along with disruption in the continuity of the retinal pigment epithelium (RPE) in the left eye at the fovea. An examination of the medical records revealed an old fundus fluorescein angiogram, from 10 years ago, which showed prominent areas of choroidal hyperfluoresence due to extensive window defects, indicative of RPE damage (Fig. 1e, f). An electro-retinogram of the patient noted a flat response in both eyes on scotopic stimulation.

## Discussion

Potassium or sodium iodate finds widespread usage in the iodination of salt. A retino-toxic dose of 20–40 mg/kg has been estimated in animal experiments [3]. The toxicity of iodate is dose dependant with a preferential involvement of the central retina [4]. In our case, although we were unable to calculate the ingested dose, we noted a peripheral rim of normal retina which might have been associated with the lower dose ingested.

The first case of retinal iodate toxicity was reported with an anti-bacterial agent Septojod in 1926. Septojod, introduced in 1920, was a German iodine preparation which contained sodium hypoiodate and sodium hypoiodide. It was thought to liberate nascent iodine upon contact with septic material. Schimmel and Riehm reported two cases of blindness following Septojod injection for puerperal septicemia. The fundus changes were noted a few days following the injection along with widespread pigmentary retinopathy. The iodate component of Septojod was blamed for the retinal changes observed [5].

Singalavanija et al. have reported a case series of 5 patients who presented with retinal toxicity of potassium iodate. They treated their patients with prednisolone, vitamin B1, B6 and B12 and reported that visual recovery depends on the amount of iodate absorbed [6]. Our case compares well with the patients described in the case series above showing outer retinal damage and atrophy but presented to us 10 years following ingestion of the inciting agent. Since a long time period had lapsed between the ingestion of the iodate and the presentation we were unable to trace the dose of the ingested agent.

Animal models on iodate retinal toxicity show histopathological changes similar to that observed in cases of dry age related macular degeneration (ARMD) with loss of the RPE and photoreceptors. Mouse models on injecting iodate have shown an upregulation of caspases at the cellular level. This leads to caspase dependant apoptosis of the photoreceptors. There is also evidence of caspase independent necrosis of the retinal pigment epithelium [7]. Further research in this field may provide valuable insight in ARMD management.

## Conclusion

Potassium iodate can produce retinal toxicity by damaging the retinal pigment epithelium and the photoreceptors cells. The degree of damage is dose-dependent but is stationary and non-progressive.
